# Inhibition of the Biofilm Formation of Plant *Streptococcus mutans*

**DOI:** 10.3390/ph17121613

**Published:** 2024-11-29

**Authors:** Gayane A. Atazhanova, Yana K. Levaya, Karakoz Zh. Badekova, Margarita Yu. Ishmuratova, Marlen K. Smagulov, Zhanna O. Ospanova, Elina M. Smagulova

**Affiliations:** 1 School of Pharmacy, Karaganda Medical University, Gogol Street, 40, Karaganda 100012, Kazakhstan; atazhanova@qmu.kz (G.A.A.); smagulovae@qmu.kz (E.M.S.); 2 Research Park of Biotechnology and Eco-Monitoring, Karaganda Buketov University, Universitetskaya Street, 28, Karaganda 100026, Kazakhstanmarlenkemel@mail.ru (M.K.S.); 3 Department of Childhood Diseases, Kazakh National Medical University Named After S.D. Asfendiyarov, Tole bi 94, Almaty 050000, Kazakhstan; ospanova.zh@kaznmu.kz

**Keywords:** biofilm formation, *Streptococcus mutans*, plants, dental caries, microbial ecology

## Abstract

This review is devoted to a systematic analysis of studies aimed at investigating plant extracts, essential oils and phytochemical compounds capable of inhibiting *Streptococcus mutans* biofilm formation. This paper investigates the effect of extracts, essential oils and individual plant compounds on inhibiting the biofilm formation of *Streptococcus mutans*, one of the major pathogens responsible for the development of dental caries. Using cultural microbiology and molecular biology techniques, the authors describe the mechanisms by which plant samples reduce *Streptococcus mutans* adhesion and growth. The results show that several plant components have antibacterial properties, contributing to the reduction of *Streptococcus mutans* colony numbers and inhibiting the synthesis of extract-exopolysaccharide matrices required for biofilm formation. This work highlights the potential of botanicals in inhibiting *Streptococcus mutans* biofilm formation, which can be applied as natural antimicrobial agents in the prevention and treatment of dental diseases. Views on the use of these plant extracts and their components in dental preparations such as toothpastes, rinses and gels aimed at preventing dental caries are evaluated. The review shows the relevance of the research to optimizing the use of plant extracts, essential oils, individual compounds and their active actions in the control of *Streptococcus mutans* biofilms.

## 1. Introduction

According to the World Health Organization’s 2022 report, about 3.5 billion people worldwide suffer from oral diseases. Approximately 2 billion people face permanent tooth decay and around 530 million children face decay of baby teeth. Increasing prevalence of these diseases is expected to fuel the demand for the dental infection control products market. The global oral care products market size is valued at USD 33.7 billion in 2021 and is expected to grow at a compound annual growth rate of 6.4% from 2022 to 2030. Increasing prevalence of dental caries among both adults and children is expected to drive the market growth [1].

Dental caries is a costly infectious disease caused by the bacterium *Streptococcus mutans* (*S. mutans*) that affects a significant proportion of the population [2]. This bacterium is the primary causative agent of tooth decay, causing the disease in 60–80% of children and virtually all adults. *S. mutans* often leads to the development of oral infections because it actively utilizes food sucrose to synthesize exopolysaccharides that play a key role in plaque accumulation, adhesion and matrix formation [3]. These processes often lead to serious infections. The ability of this bacterium to form a biofilm on host tissues is an important step in the development of infection, so limiting the population of *S. mutans* or reducing biofilm formation are considered as methods of caries prevention.

The caries treatment segment dominated the market in 2020 with a revenue share of over 35.0% [1]. Current approaches to eradicate dental biofilm include its mechanical removal and use of non-specific broad-spectrum antibiotics. Removal of bacterial biofilms during brushing requires frequent repetition because tooth surfaces are rapidly repopulated. Similarly, antimicrobial agents in mouthwashes such as CHX and delmopinol lack selectivity, affecting both pathogenic species and commensal beneficial species and causing undesirable side effects such as vomiting, diarrhea, habituation or tooth discoloration [4].

Natural products derived from medicinal plants have shown themselves to be a rich source of biologically active compounds, many of which have formed the basis for the development of new dental therapeutic and prophylactic agents. With the increasing resistance of pathogens to modern therapeutic agents such as antibiotics and antivirals, interest in the search for new antibacterial herbal remedies has increased.

The practical significance of these plant substances is conditioned by the necessity to introduce into the country a new generation of natural dental products with high antimicrobial activity and the ability to inhibit the formation of *S. mutans* biofilms.

For a comprehensive literature review, we analyzed the published data available through several search engines such as ^®^Web of Science, ^®^Scopus, PubMed and Google Scholar, using “plant extracts”, “biofilm inhibition”, “*Streptococcus mutans*”, “antimicrobial activity” and “inhibition of biofilm formation by plant *Streptococcus mutans*” as search keywords. In the available literature, there is only one review [5] which describes the properties of individual compounds isolated from plants and the inhibition of the biofilm formation of *S. mutans*; there are no reviews on the properties of extracts and EOs of plants. In this connection, we would like to present new data on these objects in a more complete manner.

## 2. Mechanism of Inhibition of *S. mutans* Biofilm Formation for Plant Samples

Oral biofilm is a complex structure of microorganisms that inhabit the oral cavity. If not removed, it can mature and cause dental caries. *S. mutans* bacteria play a key role in the formation of this biofilm, increasing its resistance to the immune system and antimicrobial agents [6]. These bacteria are the main causative agent of dental caries, forming complex structures on tooth enamel and mucosa [7].

The mechanism of inhibition of *S. mutans* biofilm formation for plant objects is a complex process involving the interaction of plant extracts with various molecular and cellular mechanisms of bacteria [8]. The essence of this process can be characterized by several main directions [9,10,11].

### 2.1. Inhibition of Bacterial Adhesion to the Surface

The main step in biofilm formation is the adhesion of bacteria to a surface, whether it is a tooth or plant material. Natural compounds from plant extracts can disrupt this step in several ways, as follows:

- Anti-adhesive action: Phenolic compounds such as catechins from green tea can block receptors on the surface of bacteria that are involved in primary adhesion. This reduces the ability of *S. mutans* to bind to the surface.
- Competition for surface sites: some plant extracts, such as extracts from citrus fruits, can act as anti-adhesive agents by competing with bacteria for attachment sites on the surface.

### 2.2. Inhibition of EPS Synthesis

EPS are polysaccharide matrices that form the backbone of a biofilm and ensure its structural integrity. These molecules play a key role in the attachment and stabilization of bacteria in the biofilm. Plant extracts can inhibit EPS synthesis through several mechanisms, as follows:

- Direct inhibition of EPS synthesis: Some components of plant extracts, such as flavonoids and tannins, can inhibit enzymes involved in exopolysaccharide synthesis such as glucosyltransferases. This results in decreased biofilm matrix formation.
- Disruption of glucosyltransferase activity: Gtfs are enzymes responsible for the synthesis of polysaccharides such as glucan. Studies show that extracts from certain plants, such as from *Punica granatum* (pomegranate), can reduce the activity of these enzymes, thereby reducing biofilm formation.

### 2.3. Disruption of Cell Signaling

Bacteria, including *S. mutans*, use cell signaling systems (e.g., through molecules such as autoinducers) to coordinate biofilm formation. These systems rely on interactions with small-molecule compounds that can stimulate or inhibit the expression of the genes responsible for biofilm formation. Plant extracts can affect this cell signaling in the following ways:

- Interference with QS signaling: Cell signaling in *S. mutans* is based on the QS process, which regulates the expression of genes responsible for biofilm formation. Plant extracts such as flavonoids can block or alter the activation of the QS system by inhibiting the expression of genes that regulate EPS synthesis and other biofilm elements.

### 2.4. Cell Membrane Damage

Plant objects such as EOs or flavonoids can penetrate the bacterial cell membrane, disrupting its integrity and permeability. This can lead to the loss of cellular contents and bacterial death. Also, such compounds can disrupt the cellular adaptation mechanisms that bacteria use to form biofilms.

- Increased membrane permeability: Phenolic compounds can interfere with the cell membrane, compromising its integrity, which weakens the ability of bacteria to form biofilms. This can affect both the physical structure of the biofilm and its ability to grow stably.

### 2.5. Genetic Changes

Natural substances can affect the expression of genes that regulate biofilm formation. This occurs through changes in the activity of various regulatory pathways, e.g.,:

- Decreased expression of genes associated with biofilms: some plant extracts can suppress the expression of genes such as gtfB, gtfC and gtfD, which are responsible for the synthesis of exopolysaccharides, or genes regulating cell aggregation.
- Antimicrobial peptide signaling activity: some plant extracts can activate regulatory pathways associated with the formation of antimicrobial peptides that inhibit bacterial growth and biofilm formation.

### 2.6. Oxidative Stress and Antioxidant Activity

Many plant extracts have antioxidant activity that can interfere with bacterial resistance and their ability to form biofilms.

- Mechanism of oxidative stress: Phenolic compounds such as polyphenols from green tea can cause the formation of free radicals that damage cellular components including membranes, DNA and proteins. This weakens the ability of bacteria to form biofilms and their resistance to external influences.

## 3. Test Methods for Plant Objects Inhibiting *S. mutans* Biofilm Formation

In recent years, there has been an increasing interest in the research and development of new antimicrobial agents from various sources to combat microbial resistance. Therefore, more attention is being paid to the screening methods and evaluation of antimicrobial activity. Various methods are used to evaluate antimicrobial activity, including diffusion tests, MIC tests and in vitro biofilm evaluation. There are numerous scientific publications on methods for testing the antimicrobial activity of plants, including their ability to inhibit *S. mutans* biofilm formation. The main testing methods include the following:

### 3.1. Minimum Inhibitory Concentration (MIC) Testing

This method determines the lowest concentration of an MIC is the lowest dosage of a plant sample that stops bacterial growth. To estimate the MIC, a sample is added to a grown *S. mutans* pathogen in different dilutions to see how the growth of the microorganism changes. The MIC and MBC values of the plant samples will be determined for eight clinical isolates using the broth microdilution method in 96-well microtiter plates. First, plant samples are prepared at a concentration of 100 mg/mL in a mixture of sterile distilled water and 5% (by volume) DMSO and sterilized by filtration. Then, double serial dilutions of the extracts (final concentrations = 100–0.195 mg/mL) in 100 μL of tryptic soy broth (TSB), supplemented with 0.5% glucose, are prepared in 96-well polystyrene microtiter plates.  After that, 100 μL of fresh bacterial suspensions (18 h, 10^7^ CFU/mL) prepared in sterile TSB, supplemented with 0.5% glucose, are added to each well. The microtitration plates are incubated for 24 h at 37 °C. The MIC values represent the lowest concentrations of plant samples at which bacterial growth will be completely inhibited. To determine the MBC values, aliquots of 10 µL each are aseptically extracted from wells in which no visible growth has been recorded and seeded onto TSB agar dishes, after which these dishes are incubated for 48 h at 37 °C. The values are considered to be the lowest concentrations at which no colony growth is observed. Each experiment is performed in three repetitions using independently grown cultures [12].

### 3.2. Kirby–Bauer Diffusion Test for Microbiologic Sensitivity

This is performed by transferring a standardized pure culture of *S. mutans* to a sensitivity dish (Petri dish with Mueller–Hinton agar) and observing growth in the presence of discs containing plant samples. The essence of the method is that paper disks impregnated with samples are placed on the surface of a dense nutrient medium seeded with a strain of *S. mutans*, and after 18–20 h, the diameters of the growth retardation zones around the disks are measured. For example, the antimicrobial effect of *Origanum vulgare* extract against *S. mutans* is analyzed (700611). First, microorganisms are reactivated and resuspended in TSB; two to three colonies are inoculated, and the inoculum is adjusted to 0.5 McFarland standard, equivalent to 1 × 10^6^ CFU/mL. An antimicrobial assay is then performed by disco-diffusion (Kirby–Bauer method) six times, and 100 mL of the corrected inoculum is placed on Mueller–Hinton agar nutrient medium. Five serial concentrations of extract [2% to 0.02% *m*/*o*] are evaluated and incubated at 37 °C for 24 h. Inhibition halos are measured with a caliper and the results are compared with a positive control of chlorhexidine 2% *w*/*v* and a negative control of ethanol [13].

### 3.3. Microtiter Method (96-Well Plate Method)

This method is used to assess the activity of samples against biofilms in microtiter plates. The microtiter (or microdilution) method for the determination of *S. mutans* activity in plant samples involves the use of dilutions in liquid medium to assess microbial sensitivity or numbers. First, a bacterial culture of *S. mutans* is prepared in an appropriate medium (e.g., TSB with added sucrose). The bacteria are incubated at 37 °C under aerobic conditions to obtain an active culture. Next, the plant samples are diluted. Next, 100 µL of sample is added to each row in each well in a microtiter plate (96-well plate). A total of 100 µL of *S. mutans* bacteria (prepared culture) is added to each well so that the final concentration is about 10^5^–10^6^ CFU/mL. The plate is incubated in a thermostat at 37 °C for 24–48 h depending on the task at hand. Bacterial growth is then assessed visually (darkening or a change in transparency) or using a spectrophotometer at 600 nm (to measure the turbidity of the culture). A method of adding dyes (e.g., resazurin or tetrazolium) that change color depending on the viability of the bacteria can also be used. Next, the MIC for the plant sample that results in significant growth inhibition of *S. mutans* is evaluated. The results are compared with control wells in which no plant samples have been added [14].

### 3.4. Biofilm Analysis Using the Crystal Violet Staining Method

This method assesses the quantity of *S. mutans* biofilm on a surface based on staining intensity. To evaluate the impact of plant extracts on *S. mutans* biofilm formation, the *S. mutans* strain UA159 preserved in skim milk is thawed, and 10 μL of the suspension is inoculated into vials containing 990 μL of Todd Hewitt broth (TBH). These vials are incubated anaerobically at 37 °C for 18 h. For purity verification, the initial suspension is streaked onto Columbia agar plates supplemented with 7% sheep blood, which are then incubated anaerobically at 37 °C for 48 h.

After the 18 h incubation, the optical density of the starter cultures is adjusted by a 1:5 dilution and measured using a MRX™ microplate reader spectrophotometer (Dynex Technologies, Chantilly, VA, USA) at 630 nm. TBH, with and without 1% sucrose, is added to 24-well flat-bottom polystyrene tissue culture plates, with a final liquid volume of 1 mL per well.

Plant sample stock solutions (100 mg/mL) are prepared in DMSO and further diluted to final concentrations of 2, 4, 6, 8 and 10 mg/mL. DMSO is added to the wells at corresponding concentrations. A 1:100 dilution of the *S. mutans* starter culture is introduced into the wells, and the plates are incubated anaerobically (95% N_2_, 5% CO_2_) at 37 °C for 24 h.

After incubation, the wells are washed with distilled water to remove loosely bound cells. The biofilm at the bottom of the wells is fixed with 95% ethanol, stained with 0.01% crystal violet solution, and the bound dye is subsequently extracted using a 33% acetic acid solution. Finally, the optical density of the extracted dye is measured at 595 nm using a MRX™ microplate reader spectrophotometer (Dynex Technologies, VA, USA) [15].

### 3.5. The Confocal Laser Scanning Microscopy Method Is Used to Visualize and Analyze the Structure of S. Mutans Biofilms

Confocal laser scanning microscopy (CLSM) is a powerful tool for studying the structure and morphology of biofilms, including those formed by bacteria such as *S. mutans*. This technique provides high-quality and high-resolution images, which allows the thickness, density and distribution of cells in biofilms to be analyzed, which is particularly useful when investigating the interaction of bacteria with plant extracts. The use of CLSM to study *S. mutans* in the context of plant extracts allows a detailed assessment of the effect of these extracts on biofilm formation and structure, as well as the visualization and analysis of microorganisms in three-dimensional space. A strain of *S. mutans* (e.g., ATCC 25175) that is capable of biofilm formation is selected for the study. The bacterial culture is grown in liquid nutrient medium containing sucrose (to stimulate biofilm formation). Plant samples (extracts, EOs and individual compounds) are prepared. The samples can be prepared in different concentrations to test their antimicrobial activity. Bacteria are inoculated on surfaces (e.g., glass slides, polystyrene 96-well plates or other suitable materials) to form a biofilm. Add 100–200 µL of bacterial culture to the medium and incubate for 24–48 h at 37 °C for biofilm formation, and plant samples into different wells with biofilm to test their effect on biofilm structure. Control wells without extract can be used to assess baseline biofilm formation. To obtain good CLSM images, bacterial cells and biofilms need to be stained to make them visible. A combination of fluorescent dyes that label different components of the biofilm can be used. After incubation with plant extracts and biofilm formation, remove excess liquid from the wells or surface. The biofilm should be stained with selected fluorescent dyes. For example, use SYTO 9 to stain live cells and PI for dead cells. Samples are stained for 15–30 min. Samples are washed to remove excess dyes. A confocal laser scanning microscope is used to obtain high-resolution images. Confocal microscopy can be used to assess the distribution of cells within the biofilm, its thickness and the interaction of cells with plant samples. This will provide insight into how they influence the biofilm morphology. Using image analysis software (e.g., ImageJ or dedicated confocal microscopy software), it is possible to quantify various parameters such as biofilm density, live-to-dead cell ratio, biofilm volume, number of cells in a particular area, etc. If the plant sample is effective in suppressing biofilm formation, we can see a smaller number of cells or a thinner biofilm and a lower density of live cells within it. For samples that have no significant effect on biofilm, the structure and density of the biofilm will remain similar to the control. Dead cells labeled with PI can be used to assess cell damage resulting from exposure to plant samples [16].

### 3.6. Method for Testing Antibacterial Activity Using a Biofilm Culture

This allows us to evaluate the ability of samples to inhibit *S. mutans* biofilms under conditions close to natural conditions, as well as to assess the effect of plant substances on biofilm formation and development, as well as on the resistance of bacteria in the biofilm. This is particularly important because bacteria in biofilms are more resistant to antibacterial drugs, making this research key to the development of new therapeutics. The stages of the test method are as follows: a strain of *S. mutans* (e.g., ATCC 25175) that is capable of forming biofilms is typically used; inoculation of the bacterium is carried out in liquid nutrient medium (e.g., TSB or Brain Heart Infusion (BHI)), supplemented with 1% sucrose to stimulate biofilm formation. Incubation is at 37 °C under aerobic or anaerobic conditions depending on the type of study. Samples are diluted to the desired concentrations for further testing (e.g., 1, 5, 10%). Surface biofilm formation is as follows: 100–200 µL of bacterial culture (with a concentration of about 10^6^ CFU/mL) is inoculated on a surface, e.g., in a 96-well microtiter plate or on glass slides, and incubated at 37 °C for 24–48 h for biofilm formation. It is important that the surface on which the biofilm forms is a suitable surface for bacteria to attach to, such as a plastic or glass surface. The control can be a well with bacteria but no plant extract (positive control with bacteria and nutrient medium) or a well with only nutrient medium (negative control). Additionally, antibiotics such as penicillin or amoxicillin can be used as a positive control to compare with the plant samples. After incubation with plant samples and antibiotics, the extent of biofilm formation should be assessed. For visualization and quantification, the crystal violet staining method (see description of this method above) can be used to measure the amount of biofilm formed in each well. It is also possible to use CLSM for a more detailed study of the biofilm structure at the cellular level [17].

Each method has its own strengths and weaknesses, and the choice of approach depends on the objectives of the study, the availability of equipment, the cost and how important detailed information on the molecular mechanisms of inhibition is. For example, imaging techniques such as confocal microscopy or staining are better suited for simple and clear analysis, while molecular and enzymatic methods provide a deeper understanding.

## 4. EOs and Their Components Inhibited *S. mutans* Biofilm Formation

Oral and dental infections, caused mainly by bacterial biofilms, are among the most common human infections worldwide. Although various antimicrobial agents can prevent biofilm formation, they often cause side effects including enamel erosion and taste disturbances.

Dental caries is considered one of the most common oral diseases worldwide with a high incidence rate in the population. It is a chronic infectious disease with multifactorial etiology that leads to the destruction of dental tissues. Due to their antimicrobial, anti-inflammatory, antifungal and antioxidant properties, plant EOs are incorporated into dental products for the prevention of oral infectious diseases. It has been demonstrated in vitro that EOs have a marked inhibitory effect on the formation of *S. mutans* EPS and their adhesion to tooth surfaces.

Plant EOs show good inhibition of *S. mutans* growth and pathogenesis. For example, an antimicrobial gel with *Melaleuca alternifolia* EO was developed that was effective against *S. mutans* biofilm in the oral cavity, reducing the bleeding index of gums [18]. Mouthwashes with this EO also reduced not only the number of *S. mutans* but also the total number of bacteria in the oral cavity [19].

In another work, peppermint EOs were loaded into chitosan nanogel for use as an antibiofilm agent against *S. mutans* and to protect its plaque, proving their potential use as an antibiofilm agent in toothpastes or rinses [20]. This study demonstrated that this nanogel promised an effective nanoformulation, with the highest inhibitory effect against some glycosyltransferase genes (gtfB, C and D) as important enzymes involved in extracellular polymers.

In a recent clinical study, an EO-based mouthwash (Listerine Antiseptic) was shown to be significantly more effective than an amine fluoride/tin fluoride mouthwash (Meridol) in inhibiting supragingival plaque formation [21].

Toothpastes containing EOs of clove, clove and oregano or clove, oregano, thyme and cinnamon were able to completely destroy *S. mutans* biofilms without differing from the control sample. Thyme EO was found to act synergistically with CHX against *S. mutans* [22].

The benefit of the association of EOs with xylitol in mouthwash against *S. mutans*-derived biofilms has also been demonstrated, regardless of the type of treatment or the age of the biofilm. It is a very promising treatment for the treatment and prevention of dental caries [23]. The EO of *Mentha piperita* and *Rosmarinus officinalis* have been shown to be effective against *S. mutans*, one of the major causative agents of dental caries. Of the peppermint EOs, menthol concentration below 3.6% was more effective than rosemary oil (containing piperitone as the main component) and CHX (at 4000 and 8000 ppm). The use of toothpaste mixed with EOs showed that lower concentrations of EOs were more effective than CHX [24]. The combination of CHX with EOs has been shown for better antibiofilm activity in oral treatment [25].

A good antibiofilm and anti-caries effect comparable to that of CHX (0.12%) was observed for a mouthwash containing *Matricaria chamomilla* L. EO (PerioGard-Palmolive). The antibiofilm effect was evaluated as a reduction in colony-forming units (CFUs) for the total *S. mutans*, *S. sobrinus* and *Lactobacillus* sp., and the anti-caries effect was studied as an effect on enamel demineralization compared to phosphate buffer solution. The authors found a 39.4% reduction in mineral loss in the case of mouthwash containing *Matricaria chamomilla* L. EO, which is very close to that of CHX (47.4%). The authors considered that the experimental product containing chamomile EO significantly reduced enamel demineralization [26].

Mouthwashes often include ingredients like CHX, fluoride, delmopinol chloride, cetylpyridinium chloride and alcohol. While these components are effective in preventing oral diseases, there is growing concern among patients about the frequent use of such chemicals. As a result, many patients seek mouthwashes formulated with natural ingredients [27]. In this context, essential oils (EOs) offer a promising alternative for creating effective, natural-based mouthwashes—mouthwashes containing *Citrus hystrix* leaf EO alone or in combination with CHX-inhibited periodontopathogenic bacteria and biofilms of *S. sanguinis* and *S. mutans* [28].

**Figure 1:**
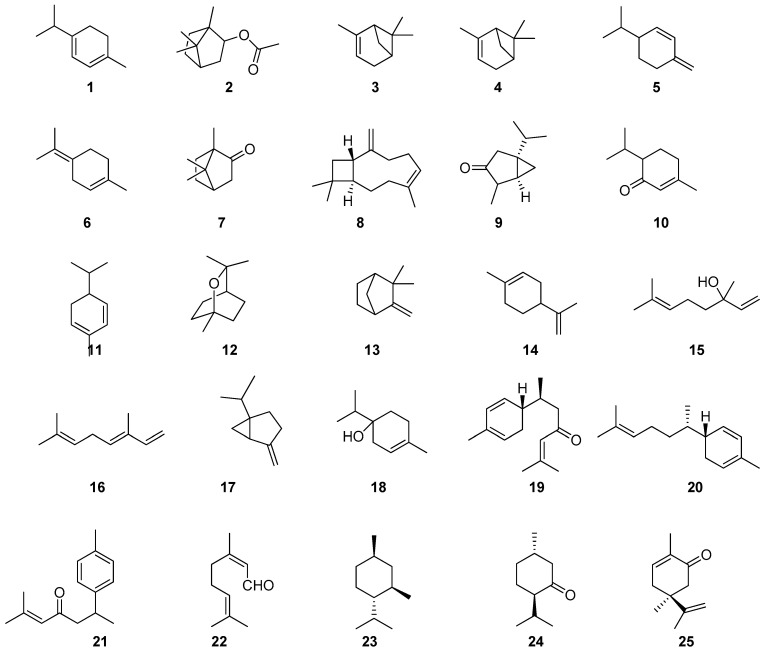
Chemical compounds of plant EOs inhibiting *S. mutans* biofilm formation.

**Figure 2:**
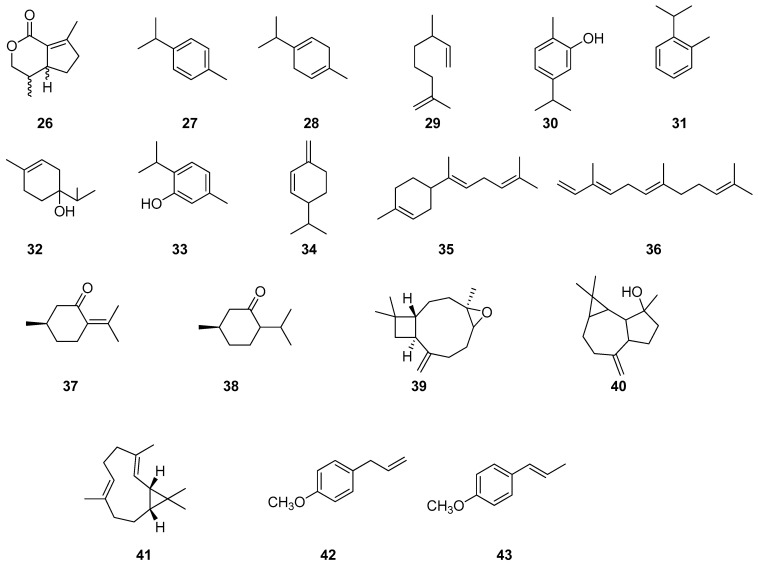
Chemical compounds of plant EOs inhibiting *S. mutans* biofilm formation.

As can be seen from Table 1, many EOs and their components have anti-caries activity and are substances for the creation of antibacterial preparations and prophylactics on their basis. For example, the EO of *C. aurantium* L. [29] can be used as an additive for mouthwash because it shows great potential, even at a low concentration of *C. aurantium* L. The EO effectively reduces bacterial growth and destroys mature biofilms. Figure 1 and Figure 2 present the main chemical compounds of the plant EOs 1–43 related to the inhibition of the biofilm formation of *S. mutans*.

EO from the rhizome of *Etlingera pavieana* in Thailand is used to control streptococci including *S. mutans* and *S. sobrinus*. The main components of the EO are methylchavicol **42** and trans-anethol **43**.

The isolated methylhavicol **42** showed almost pronounced antibiofilm activity against *S. mutans* and *S. sobrinus* and was recommended as an active compound for antistreptococcal activity [46]. Another study evaluated the antibacterial activity of terpinen-4-ol monoterpenoid **32** against *S. mutans* and *Lactobacillus acidophilus* and its effect on gbpA (*S. mutans*) and slpA (*L. acidophilus*) gene expression [47]. The results demonstrate the antimicrobial activity of terpinen-4-ol **32** and its ability to modulate gbpA and slpA gene expression, emphasizing the therapeutic ability of terpinen-4-ol **32** as an alternative to inhibit adhesion in biofilms. Researchers found that the sesquiterpene β-caryophyllene **8** could inhibit cariesogenic biofilm and could be a potential agent for the prevention of dental caries [48]. Another study [49] evaluated the effect of EO on *Lippia origanoides* of different chemotypes on the inhibition of *S. mutans* biofilm. *L. origanoides* chemotypes carvacrol **30** + thymol **33** and thymol **33** showed a pronounced reduction in topography, microbial cell number and extracellular matrix on polymicrobial biofilm.

EOs come from natural sources, making them attractive for use in natural medicine and alternative therapies. This may be important for those who prefer to avoid synthetic antimicrobial agents. Plant EOs can act through a variety of mechanisms including disruption of the cell membrane, suppression of EPS synthesis and inhibition of cell signaling to effectively reduce the ability of *S. mutans* to form a biofilm. Unlike synthetic antibiotics, which can induce the development of resistance in microorganisms, EOs tend to reduce the likelihood of resistant strains because their active components affect multiple cellular targets. Some EOs (e.g., *Mentha spicata* and *Eucalyptus globulus*) are used in dental products, such as pastes and rinses, and may not only inhibit biofilm formation, but may also have a positive effect on gum health by reducing inflammation and preventing periodontitis [35]. But there are the following non-disadvantages of EOs in inhibiting *S. mutans* biofilm formation: high toxicity if misused, lack of standardized dosages and possible allergic reactions [50]. Although EOs show promising results in laboratory studies, their clinical efficacy in inhibiting *S. mutans* biofilm is still poorly understood. Many studies are limited to in vitro tests, and more data are needed to confirm their effects in real oral conditions. 

Thus, EOs have traditionally been used in medicine because of their biological activity. They may contain substances that inhibit bacterial growth and biofilm formation, making them promising candidates for the development of new agents for the prevention of dental infections.

## 5. Plant Extracts Inhibited Biofilm Formation of *S. mutans*

Biofilm formation by *S. mutans*, a Gram-positive bacterium, on tooth enamel is a major cause of plaque and dental caries [49]. *S. mutans* has the ability to utilize sucrose from consumed food as a factor in biofilm formation. Many enzymes are involved in the biofilm formed by *S. mutans*. These include sucrose phosphorylase, glucosyltransferase and fructosyltransferase. These enzymes can utilize sucrose to synthesize water-insoluble glucans and fructans for attachment and biofilm formation of *S. mutans*. The bacterium synthesizes an insoluble glucan layer that includes gtf, which accumulates in dental plaque and contributes to dental caries formation. The virulence factors of *S. mutans* associated with cariesogenicity are a set of gtf genes. Gtf synthesizes glucan polymers from sucrose, which provide binding sites for bacterial adhesion on tooth surfaces and contribute to plaque formation.

The use of biofilm inhibitors, such as sodium fluoride, against *S. mutans* can effectively prevent its biofilm formation (Table 2), but high levels of sodium fluoride can be toxic. The use of natural products to inhibit biofilm formation, such as extracts from many plants, has been proposed.

Plant extracts, like EOs, have antimicrobial activity and can inhibit the biofilm formation of *S. mutans*. Plant extracts such as green tea, pomegranate, garlic, turmeric and others have proven antimicrobial activity against *S. mutans* [80,81,82,83,84]. These extracts can effectively inhibit the growth of bacteria and their ability to form biofilms; for example, green tea extract contains catechins, which have potent antioxidant and antimicrobial activity. Studies have shown that green tea extracts can inhibit biofilm formation by reducing the amount of EPS secreted by *S. mutans* [80]. Pomegranate extract contains tannins and anthocyanins, which have antimicrobial activity and can inhibit bacterial adhesion to tooth enamel, as well as reduce bacterial aggregation in biofilms [81]. Turmeric extract has anti-inflammatory and antimicrobial properties. It is able to inhibit the synthesis of exopolysaccharides, which prevents the formation of biofilm [82]. Garlic extract contains allicin, which exhibits potent antimicrobial activity, including the inhibition of biofilm formation and destruction of bacterial membranes [83]. Black walnut extract is used to destroy pathogenic microorganisms and can inhibit the growth of *S. mutans*, reducing the likelihood of biofilm formation [84].

Plant extracts have significant potential in inhibiting *S. mutans* biofilm formation, but their use requires caution. Advantages include naturalness, diversity of active components and lack of stability, but important limitations include problems with dosage, stability and possible toxicity with long-term use. Further research is recommended to better understand the mechanisms of action of the extracts and their clinical effectiveness.

## 6. Individual Components Isolated from Plants That Inhibit *S. mutans* Biofilm Formation

Suppressing the growth and biofilm formation of *S. mutans* is one of the strategies to prevent dental caries. Although several anti-plaque agents have been used, attempts to find an effective agent are still ongoing. Individual plant compounds such as flavonoids, phenolic compounds and others can inhibit *S. mutans* biofilm formation through their antimicrobial activity and ability to interfere with the mechanisms that promote biofilm formation. These compounds can be used in different concentrations to achieve optimal effects. For example, some studies have shown [64,65,66,67,68,69,70,71,72,73,74,75,76,77,78,79,80,81,82,83,84,85] that several individual compounds isolated from plant material such as *Aralia cachemirica*, *Libidibia ferrea*, *Withania somnifera*, *Piper betle*, etc. have demonstrated a plaque-inhibitory effect.

Phenolic compound thymol **33** at a concentration of 50–150 µg/mL disrupts the integrity of the bacterial cell membranes, which reduces their ability to adhere and form a biofilm [49].

**Figure 3:**
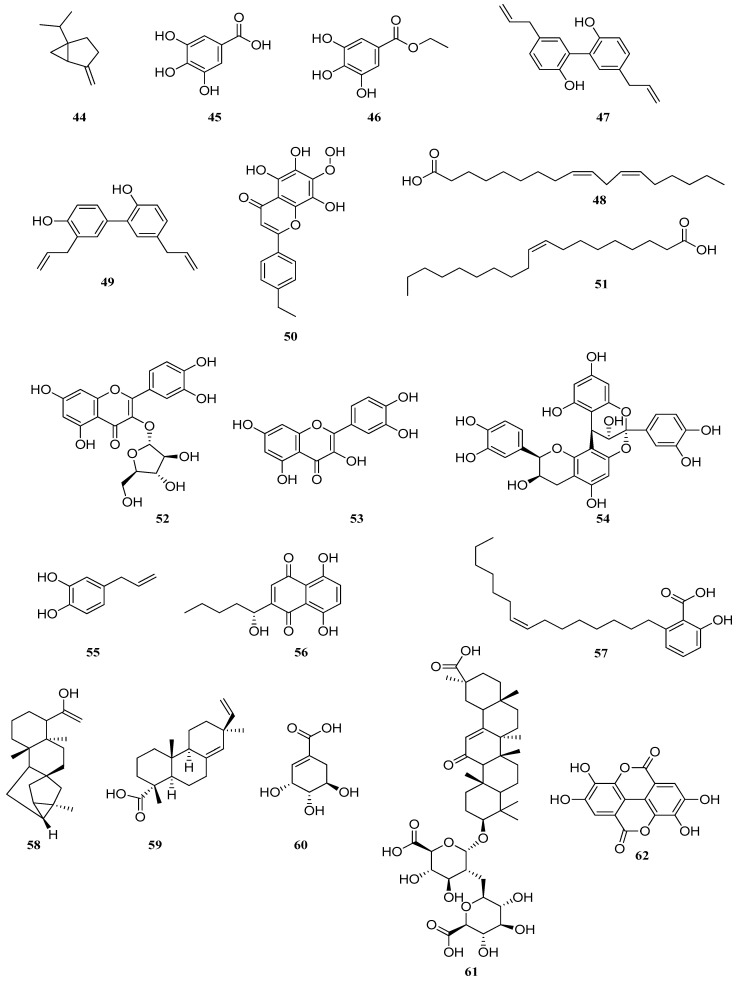
Chemical compounds isolated from plants that inhibit *S. mutans* biofilm formation.

Monoterpene, sabinene **44** (Figure 3) suppresses the growth, biofilm formation and adhesion of *S. mutans* by inhibiting cariesogenic virulence factors. Sabinene **44** suppresses biofilm formation by inhibiting gtfB, gtfC, gtfD and vicR, and suppresses acid production by inhibiting the expression of brpA and relA, and *S. mutans* adhesion was significantly inhibited by 0.1–0.2 mg/mL of sabinene [85].

The isolated gallic acid **45** and ethyl gallate **46** from *Libidibia ferrea* (Mart. ex Tul.) LP Queiroz extract decreased viable cells, alkali-soluble glucans, acidogenicity and acidity, downregulated the expression of gtfB, gtfC and gtfD genes in biofilms and showed decreased biomass, exopolysaccharide and microcolonies of *S. mutans* [65].

In a study [86], the antibacterial activity of magnolol **47** and honokiol **48**, the main components of magnolia plant bark, against planktonic cells and *S. mutans* biofilm was studied and compared with that of CHX. The MIC of magnolol **47**, honokiol **48** and CHX for *S. mutans* was 10, 10 and 0.25 μg/mL, respectively. In addition, each agent showed bactericidal activity against *S. mutans* planktonic cells and inhibited biofilm formation in a dose- and time-dependent manner. Magnolol **47** (50 μg/mL) had greater bactericidal activity against *S. mutans* biofilm than honokiol **48** (50 μg/mL) and CHX (500 μg/mL) after 5 min of exposure, while all of them showed weak activity against the biofilm after 30 s. Magnolol was found to have antimicrobial activity against the planktonic and biofilm cells of *S. mutans*. Magnolol may be a candidate for the prevention and treatment of dental caries.

The flavone 5,6,8-trihydroxy-7-methoxy-2-methoxy-2-(4-methoxyphenyl)-4H-chromen-4-one **49** obtained from *Dodonaea viscosa* var. angustifolia shows antimycobacterial activity against *S. mutans*, and antibiofilm and anti-acidogenic activity, so it can be used in the oral cavity to prevent dental caries [87].

It was found that treatment with linoleic **50** and oleic **51** acids during biofilm formation also inhibited biofilm accumulation and the rate of acid production by the biofilm without killing the biofilm bacteria [88].

Quercetin-3-arabinofuranoside **52**, myricetin **53** and procyanidin A2 **54** showed the greatest inhibition of *S. mutans* virulence traits; the combination of these compounds showed enhanced effects [89].

The mechanism of inhibition of another polyphenolic compound 4-allylpyrocatechin **55** was bactericidal and fungicidal effects. The compound potentially inhibits the growth and biofilms of oral *Streptococcus* and *Candida* spp., with different mechanisms of action and kill rates [90].

Shikonin **56** is extracted from the roots of *Lithospermum erythrorhizon*. The compound showed marked activity against *S. mutans* and inhibited the growth and biofilm formation of this bacterium. In a clinical study in which 20 subjects were asked to brush their teeth for 1 week, toothpaste containing shikonin reduced the number of *S. mutans* in the oral cavity, whereas no such effect was observed after using toothpaste without shikonin [91].

Ginkgoneolic acid **57**, derived from *Ginkgo biloba*, demonstrated inhibitory effects on *S. mutans*. It suppressed the growth of planktonic cells with MIC of 4 μg/mL and MBC of 8 μg/mL. At sub-MIC levels, the compound effectively reduced acid production and *S. mutans* adhesion to saliva-coated hydroxyapatite. Additionally, ginkgoneolic acid significantly inhibited *S. mutans* biofilm formation (MIC_50_ = 4 μg/mL) and reduced established 1-day biofilms by over 50% at a concentration of 32 μg/mL (MBRC_50_) [92].

Ent-trachyloban-19-oic acid **58**, isolated from *Iostephane heterophylla*, inhibited the growth of *S. mutans* at concentrations ranging from 4.1 μg/mL to 70.5 μg/mL and in this regard, the authors proposed it as a promising antibacterial agent against *S. mutans* biofilms [93].

Epi-pimaric acid **59**, derived from the aerial parts (stems and leaves) of *Aralia cachemirica* L. (*Araliaceae*), demonstrated notable activity against oral pathogens [94]. Its MIC ranged from 4 to 16 μg/mL, with the MBC being two to four times higher than the MIC. The compound significantly inhibited *S. mutans* biofilm formation on saliva-coated surfaces (*p* < 0.05). Confocal microscopy further revealed that epi-pimaric acid **59** effectively blocked the adhesion and attachment of *S. mutans*.

Shikimic acid **60** isolated from *Illicium verum* can reduce EPS synthesis and modulate the transcription of genes associated with biofilm formation. It interacts with the cell membrane and membrane proteins, causing damage to bacterial cells [95].

In [96], the inhibitory activity of glycyrrhizic acid **61** from *Glycyrrhizae Radix* and ellagic acid **62** from *Rubi Fructus* against *S. mutans* gtf activity was studied. Analysis of enzyme kinetics revealed the mechanism by which glycyrrhizin and ellagic acid inhibit enzyme activity. Ellagic acid **62** [96] and tannins at concentrations of 100–500 µg/mL inhibit the synthesis of EPS, which prevents biofilm formation. They are effective against several bacterial strains, improving oral hygiene, and can also interact with bacterial cell membranes, weakening their integrity.

The isolated compound 3,7,11,15-tetramethyl-hexadec-2-en-1-ol **63** from *Cynodon dactylon* showed higher antibiofilm activity on *S. mutans*. The MIC of *S. mutans* was at 12.5 mL [97].

**Figure 4:**
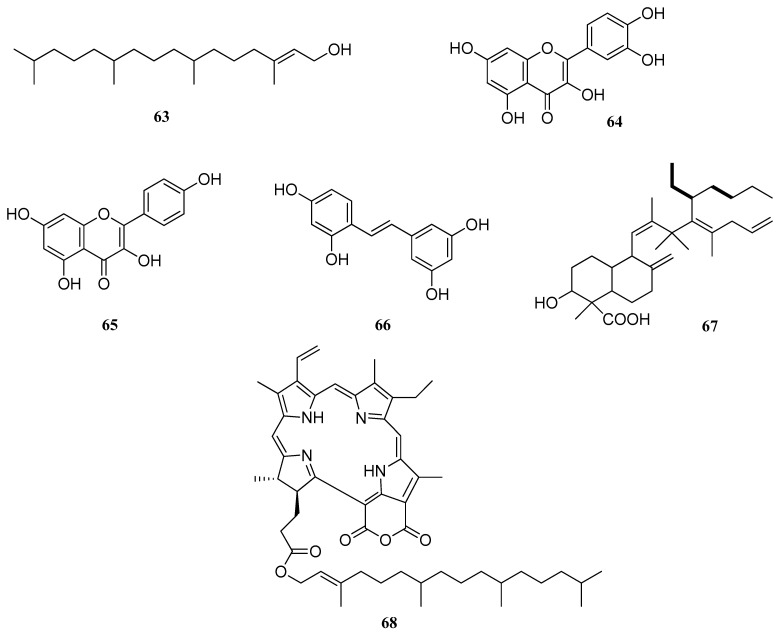
Chemical compounds isolated from plants that inhibit *S. mutans* biofilm formation.

Flavonoids such as quercetin **64** and kaempferol **65** (Figure 4), isolated from the medicinal plant *Uvaria chamae* P. Beauv, demonstrated antibiofilm activity compared to the negative control. They were able to reduce biofilm dry weight, total protein, viable cells measured by CFUs and the formation of insoluble and soluble glucans. Quercetin and kaempferol demonstrated a comparable ability to kill *S. mutans* in biofilms compared to CHX [77].

Oxyresveratrol **66**, a plant-derived natural compound, exhibits inhibitory effects against various bacterial species, including *S. mutans*. Its inhibitory action on *S. mutans* survival is dose-dependent, with a concentration of 250 μg/mL significantly reducing bacterial survival, disrupting biofilm formation and inhibiting the synthesis of water-soluble glucans. At this concentration, oxyresveratrol also markedly downregulated the expression of gtfB and gtfC genes. However, it upregulated lactate dehydrogenase activity and increased the expression of the ldh and atpD genes. Additionally, the upregulation of gtfD suggested an enhancement in the synthesis of water-soluble glucans. Stress-response genes such as vicR, liaR and comDE were also upregulated, indicating activation of self-defense mechanisms. Overall, oxyresveratrol suppressed *S. mutans* growth, reduced biofilm formation, acid production and glucan synthesis, while simultaneously activating its self-defense pathways [98].

It was found that compound **67**, isolated from the bulbs of *Myrmecodia pendans* (Merr and Perry), a native plant of Papua, is a diterpene derivative of labdane. The MBC value of the terpenoid against *S. mutans* biofilms was 50 ppm and the MBEC value for an induction time of 1 min was 40% [99].

Compound **68** was isolated from the chloroform extract of *Clinacanthus nutans* leaves. Based on spectral data, it was found to be structured as purpurin-18 68 wick ester, which significantly reduced *S. mutans* biofilm formation below 50% at 25 μg/mL (*p* < 0.05). These results suggest that purpurin-18 **68** phytyl ester may be an effective agent to inhibit the virulence of *S. mutans* biofilms [100].

Flavonoids at concentrations of 50–200 µg/mL, such as epigallocatechin gallate, inhibit the adhesion of *S. mutans* to dental enamel and also suppress the synthesis of EPS that make up the biofilm matrix [101].

Rosmarinic acid also showed significant inhibitory activity at 5 mg/mL concentration on *S. mutans* biofilm formation in 1% sucrose containing medium [102]. Thus, individual plant compounds can effectively inhibit *S. mutans* biofilm formation, but their effect depends on the concentration and properties of the specific substance. It is important to maintain optimal dosages to minimize side effects (e.g., toxicity or allergic reactions) and to take into account the possible instability of extracts in solutions. Research shows that combinations of different plant compounds can enhance their effects and provide more stable results, so optimal concentrations should be carefully selected for each specific compound.

Therefore, the discovery of new antibiofilm agents, especially from natural products, helps to develop effective strategies against this type of disease.

## 7. Conclusions

Dental caries is among the most prevalent and expensive biofilm-related infectious diseases globally. The bacterium *S. mutans* plays a key role in its development due to its capacity to produce acids and synthesize EPSs. EPSs are considered an important virulence factor of cariesogenic biofilm, increasing its resistance to antimicrobial agents and enhancing its virulence compared to single bacterial cells. Traditional caries treatment methods such as CHX and antibiotics have side effects and can cause drug resistance. With the development of modern technology, new approaches are being introduced to synthesize and discover antimicrobial agents. In this review, we examined the current antimicrobial agents (plant extracts, EOs and individual compounds) targeting *S. mutans* biofilms.

The search for alternative methods to control biofilm-related diseases is currently emphasized. The use of low-molecular-weight compounds as drugs helps to reduce development costs and risks, but there are difficulties in preventing and treating dental biofilms with these drugs due to their poor solubility, limited exposure time, salivary washout and difficulty penetrating through the EPS biofilm matrix. Nanoparticles have shown their ability to improve the penetration of low-molecular-weight compounds into biofilms, increasing the stability and bioavailability of drugs, making them promising for clinical applications. In the future, web servers and computational tools can be used to develop new methods for the prediction and synthesis of antimicrobial agents. However, one limitation of these methods is the need for standardized and reliable biological data to create effective models. In addition, it is important to note that many current studies focus on in vitro experiments or animal models with single-species biofilms, which limits their potential application in clinical practice. The mechanism of action of low-molecular-weight compounds on biofilm is not fully understood. The complex oral environment, including a variety of microbes, saliva and enzymes, may influence the in vivo efficacy of new agents. Combining techniques such as computational tools and algorithms can be an effective approach to synthesize novel antimicrobial agents. For their successful introduction into clinical practice, further research is required to better assess antimicrobial activity, biocompatibility and to find efficient drug delivery systems.

In conclusion, all the extracts, EOs and plant compounds considered exhibit significant antibiofilm activity against *S. mutans* and have potential for the development of new anti-caries agents. Natural products represent an important and cost-effective source in the search for antimicrobial agents. However, there are certain risks to their use: traditional agents such as fluoride, CHX and antibiotics are often accompanied by side effects and low selectivity, which may disturb the balance of the oral microbiome.

## Figures and Tables

**Table 1:** Plant EOs inhibiting *S. mutans* biofilm formation.

Species	Location	Number of Identified Compounds	Anti-Caries Properties EOs	Main Compounds	Reference
*Chamaecyparis obtusa*	South Korea	59	The EO of *Chamaecyparis obtusa* exhibited a concentration-dependent inhibitory effect on bacterial growth, with significant inhibition observed at concentrations above 0.025 mg/mL and achieving 99% inhibition at 0.2 mg/mL. Additionally, bacterial biofilm formation and acid production were markedly reduced at concentrations exceeding 0.025 mg/mL. Real-time PCR analysis revealed a decrease in the expression of virulence factor genes, including brpA, gbpB, gtfC and gtfD	α-terpinene **1** (40.60%), bornyl acetate **2** (12.45%), α-pinene **3** (11.38%), β-pinene **4** (7.22%), β-phellandrene **5** (3.45%), α-terpinolene **6** (3.40%)	[30]
*Chrysanthemum boreale*	South Korea	72	The EO of *C. boreale* showed significant inhibition of bacterial growth, adhesive ability and acid generation of *S. mutans* at concentrations of 0.1–0.5 mg/mL and 0.25–0.5 mg/mL, respectively. Real-time PCR analysis showed that the gene expression of some virulence factors such as gtfB, gtfC, gtfD, gbpB, spaP, brpA, relA and vicR of *S. mutans* was significantly reduced in a dose-dependent manner	camphor **7** (20.89%), β-caryophyllene **8** (5.7%), α-thujone **9** (5.46%), piperitone **10** (5.27%), sesquiphellandrene **11** (5.16%), α-pinene **3** (4.97%), 1,8-cineole **12** (4.52%), β-pinene **4** (4.45%) and camphene **13** (4.19%)	[31]
*Citrus aurantium*	Algeria	24	The EO reduced the growth of *S. mutans* and decreased the expression of comC, comD, comE, gtfB, gtfC and gbpB genes	limonene **14** (50.5%), linalool **15** (9.89%), β-ocimene **16** (7.82%), sabinene **17** (5.1%)	[29]
*Citrus limon*	China	40	The EO reduced biofilm formation by 97.87% at 1/2MIC and 1/8MIC	β-terpinene **18** (17%)	[32]
*Curcuma longa*	South Korea	42	*C. longa* EO inhibited the growth and acid generation of *S. mutans* at concentrations ranging from 0.5 to 4 mg/mL. The EO also showed significant inhibition of *S. mutans* adhesion to hydroxyapatite beads at concentrations above 0.5 mg/mL	α-turmerone **19** (35.59%), germacron **20** (19.02%), α-zingiberene **21** (8.74%)	[33]
*Cymbopogon citratus*	Brazil	21	EO at a concentration of 1.2 mg mL reduced the number of adhered cells (n = 15 for each group) by 100%	citral **22** (80%)	[34]
*Eucalyptus globulus*	Peru	9	The inhibition halo was 27.0 ± 0.82 mm and the MIC was 1.9168 mg/mL; the EO showed antimicrobial activity against both planktonic and biofilm cultures of *S. mutans*	1,8-cineole **12** (65.83%)	[35]
*Eucalyptus*	Saudi Arabia	N/M	The diluted EO showed a significant decrease in total absorption against *S. mutans* compared to the control	1,8-cineole **12**	[36]
*Hyssopus ambiguus* (Trautv.) Iljin	Kazakhstan	48	EO at concentrations of 6, 8 and 10mg/mL reduced *S. mutans* biofilm formation by 73, 98 and 98%, respectively, compared to untreated bacteria (*p* < 0.05)	1,8-cineole **12** (61.7%), α-pinene **3** (14.1%), β-phellandrene **5** (5.4)	[37]
*Juniperus excelsa* M. Bieb	Lebanon	27	XTT analysis and CFU counts confirmed that the 10-fold diluted EO determined a statistically significant (*p* < 0.05) reduction in bacterial counts and viability against both biofilm and planktonic forms	α-pinene **3** (86.8%)	[38]
*Mentha piperita*	Brazil	N/M	*M. piperita* oil significantly reduced bacterial biofilm, showed a synergistic effect when combined with CHX and did not cause cell toxicity. *M. piperita* oil destroyed pre-formed biofilms and inhibited biofilm formation at sub-inhibitory concentrations	menthol **23** (42%), menton **24** (46%)	[39]
*Mentha spicata*	Peru	19	The inhibition halo was 18.3 ± 0.47 mm, and the MIC was 1.8484 mg/mL	carvone **25** (57.93%), limonene **14** (12.91%)	[35]
*Nepeta cataria* L.	Kazakhstan	50	EO at a concentration of 4 mg/mL reduced *S. mutans* biofilm formation by 97% compared to untreated bacteria (*p* < 0.05)	1,8-cineole **12** (15.8%), (4aR,7S,7aS)-nepetalactone **26** (29.3)	[37]
*Origanum heracleoticum* L.	France	31	In comparison to the positive control Penicillin/streptomycin 100X (DIZ: 34.13 ± 0.85 mm, MIC: 0.78125 μL/mL, MBC: 6.25 μL/mL), *Origanum heracleoticum* L. (DIZ: 39.67 ± 0.81 mm, MIC: 0.625 μL/mL, MBC: 1.25 μL/mL) demonstrated similar effectiveness in inhibiting acid production and reducing the hydrophobicity and biofilm formation of *S. mutans* at concentrations ranging from 1/2 to 1 MIC	p-cymene **27** (10.37%), γ-terpinene **28** (8.74%)	[40]
*Origanum vulgare* L.	Bosnia	21	The EOs of *Origanum vulgare* L. (DIZ: 80 mm, MIC: 0.625 μL/mL, MBC: 2.5 μL/mL)	p-cymene **27** (17.32%), γ-terpinene **28** (10.67%)	[40]
*Origanum vulgare* L.	Iran	N/M	The frequency of *S. mutans* was 15.3%, 87% of which was capable of biofilm formation. The MIC of the oregano EO was 50 µL/mL against the *S. mutans* isolates, and 82% of the isolates did not grow at the concentrations of ≥512 µL/mL. However, none of the isolates were capable of biofilm formation at the MIC and sub-MIC concentrations of the EO	limonene **14** (12.065), myrcene **29** (10.812)	[41]
*Origanum vulgare* L.	Kazakhstan	21	EO at a concentration of 2 mg/mL reduced *S. mutans* biofilm formation by 99% compared to untreated bacteria (*p* < 0.05)	carvacrol **30** (65.4%), o-cymene **31** (13.2%)	[37]
*Tea* tree	China	100	The complete death of *S. mutans* bacteria was observed when the concentration of EO was higher than the MBC (0.25%) and no bacterial growth was observed for a period of 24 h. The bacteriostatic rate was similar to that of 0.2% CHX; the difference was not statistically significant (*p* ≥ 0.05)	terpinen-4-ol **32** (30%), 1,8-cineol **12** (15%), α-terpinene **1** (8%)	[42]
*Thymus zygis*	Spain	Up to 52	EO showed stronger antibacterial activity with MIC = 0.625 μL/mL; it was considered bactericidal against *S. mutans*. EO reduced biofilm formation by 98.11% and 95.74% at 2MIC and 1/2MIC	thymol **33** 30.48%, carvacrol **30** 4.85%	[43]
*Zingiber officinale*	Turkey	N/M	The MIC (21.25 µL/mL) value of ginger EO inhibited biofilm formation on the stainless-steel space maintainer by 89.16%. Ginger EO can be used to prevent dental caries by preventing biofilm formation of cariogenic *S. mutans*	β-sesquiphellandrene **34** (9.29%), bisabolene **35** (5.32%), farnesene **36** (5.20%)	[44]
*Ziziphora clinopodioides Lam*	Kazakhstan	25	EO at a concentration of 9 mg/mL reduced *S. mutans* biofilm formation by 98% compared to untreated bacteria (*p* < 0.05)	pulegone **37** 42.7%, isomentone **38** 15.3%	[37]
*Salvia dumetorum Andrz. ex Besser*	Kazakhstan	77	EO at concentrations of 4–10 mg/mL reduced biofilm formation of *S. mutans* by 95–97%	β-caryophyllene **8** 13.21%, caryophyllene oxide **39** (11.16%), spathulenol **40** (10.75%), bicyclogermacrene **41** (5.58%)	[45]

**Table 2:** Plant extracts inhibiting *S. mutans* biofilm formation.

Plant Name	Location	Solvent	Inhibition of *S. mutans* Biofilm Formation	Reference
*Aerva sanguinolenta*	Northern Gangetic Plain of West Bengal	Ethanol	Enamel exposed to 2 mL of 0.2% solution of *A. sanguinolenta* for 2 min can significantly inhibit biofilm formation and positively inhibit the underlying demineralization in the cariesogenic environment	[51]
*Állium satívum*	Korea	Freshly squeezed garlic juice	Garlic extract had a clear antibacterial effect on all microorganisms. It also increased the attachment of *S. mutans* to orthodontic wires. The low concentration of garlic extract also increased the gene expression of the gtf gene of *S. mutans*	[52]
*Artemisia princeps*	Korea	70% Ethanol	After using the safranin staining method, *A. princeps* extract was found to have an inhibitory effect on biofilm formation at a concentration of >0.05 mg/mL	[53]
*Backhousia citriodora*	Japan	Water	The extract inhibited glucansaccharose activity and also reduced the drop in glycolytic pH in the *S. mutans* culture and inhibited lactate production by at least 46%	[54]
*Caesalpinia sappan*	Chiang Mai Province (Thailand)	Ethanol extract fraction	The results showed that the fraction even at a low concentration inhibited the biofilm formation of *S. mutans*	[55]
*Camellia sinensis*	Iran	Methanol–water	Dental caries was significantly reduced at low extract concentrations, and the efficacy of semi-fermented tea was higher than that of non-fermented extracts due to the rich content of volatile components	[56]
*Camellia japonic*	Korea	Methanol	Extracts (1.0 mg/mL) significantly inhibited bacterial growth by more than 76%. The minimum inhibitory concentrations were determined as 0.5 mg/mL using a disk diffusion assay on solid agar medium	[57]
*Catha edulis*	Yemen	Water	The extracts effectively inhibited biofilm formation. The minimum biofilm inhibitory concentration varied among cultivars (0.25–1%). The extracts also inhibited glucan synthesis, especially insoluble glucans (average 85% inhibition at 1%), with significant differences between varieties	[58]
*Cichorium intybus var. silvestre*	N/M	Homogenized juice	The extracts inhibited biofilm formation from 16–62%	[59]
*Cinnamomum verum*	N/M	Water	Nicotine-induced *S. mutans* biofilm formation was reduced from 34 to 98% in the presence of 2.5 mg/mL aqueous extract of cinnamon	[60]
*Cistus creticus ssp. creticus*	Greece	Methanol	Significantly inhibited *S. mutans* biofilm formation at 0.60 mg/mL	[61]
*Cistus monspeliensis*	Greece	Methanol	The extract of *C. monspeliensis* had a strong inhibitory effect on *S. mutans* biofilm formation at a concentration of 0.60 mg/mL (mean OD_595_ = 0.106)	[61]
*Croton urucurana*	Brazil	Fractions aqueous–alcoholic, DMSO	The extract fractions demonstrated inhibition of *S. mutans* on biofilm formation in vitro	[62]
*Cymbopogon citratus*	India	Water	The extract showed the highest efficacy in reducing *S. mutans* biofilm formation and adhesion activity, achieving 90% inhibition at a MIC value of 12 mg/mL	[63]
*Emblica officinalis*	India	Ethanol	A total of 50% reduction in adhesion at concentrations of 156 mg/mL and 312.5 mg/mL of extract and ethanol fraction, respectively. Effective reduction was observed in glucan synthesis and cell-surface hydrophobicity. qRT-PCR revealed significant suppression of genes involved in its virulence	[64]
*Libidibia ferrea*	Brazil	Ethanol	Fruits and seeds extract showed marked antimicrobial activity, inhibition of adhesion and reduction in acidogenicity and acidity in *S. mutans* biofilms	[65]
*Lime leaves* extract	Thailand	Water	Leaf extract inhibited the growth of *S. mutans*, which corresponds to the activity of the antibiotic ampicillin. The formation of *S. mutans* biofilm was also inhibited by the extract	[66]
*Oenothera biennis*	Japan	Ethanol	The extract induced strong aggregation of *S. mutans* MT8148 cells, while cell surface hydrophobicity decreased by about 90% at a concentration of 0.25 mg/mL. Sucrose-dependent adhesion of MT8148 cells was also decreased upon addition of *O. biennis* seed extract, with a reduction rate of 73% at a concentration of 1.00 mg/mL. Insoluble glucan synthesis was significantly decreased when *O. biennis* seed extract was present at concentrations greater than 0.03 mg/mL. In an animal experiment, caries rates in rats given the seed extract (0.05 mg/mL in drinking water) were significantly lower than in rats given the extract	[67]
*Origanum vulgare*	Greece	Methanol	The extract significantly inhibited *S. mutans* biofilm formation at 0.60 mg/mL	[61]
*Psidium cattleianum*	Brazil	Water	Leaf extract killed *S. mutans* grown in biofilms when applied in high concentrations. At low concentrations, it inhibited acid production by *S. mutans* and reduced the expression of proteins involved in general metabolism, glycolysis and lactic acid production	[68]
*Polygonum cuspidatum*	Korea	Methanol fraction extract	At a concentration of 1.5 mg/mL, the fraction inhibited approximately 2 log(10)CFU/mL *S. mutans* after 2 h of exposure	[69]
*Rhamnus prinoides*	USA	Ethanol and water	Ethanol extracts of leaves and stems significantly inhibited *S. mutans* biofilm formation by up to 99.9% and reduced planktonic cell growth by up to 10 logarithmic units compared to untreated control samples	[70]
*Rhamnus prinoides (gesho)*	USA	Ethanol	Extract suppressed the growth of *S. mutans* planktonic cells and reduced the production of biofilm polysaccharides by up to 99%	[71]
*Rhodiola rosea*	China	Ethanol	Suppressed biofilm formation and EPS synthesis of *S. mutans*, expression of gtf genes and the quorum sensing system and enzymatic activity of gtf proteins. Moreover, the extract showed good biocompatibility with human cells	[72]
*Rhus coriaria*	Iran	Water	The extract reduced bacterial biofilm formation on orthodontic wire at MIC and 1/8 of MIC of *S. mutans* ATCC 35608 22.2–86.1%	[73]
*Rosmarinus officinalis*	Greece	Methanol	The extract at MIC: 0.08–5.00 mg/mL showed high antibacterial activity	[61]
*Salvia deserta*	China	Ethanol	The MIC of SFE against *S. mutans* was 14 μg/μL. SFE at a concentration of 1/8 MIC to MIC could inhibit the growth rate of *S. mutans* for 30 h and could significantly inhibit the LDH activity compared with the control group (*p* < 0.0001). SFE at concentrations ranging from 4 MIC to 1/4 MIC had an inhibitory effect on the acid production of *S. mutans* (*p* < 0.001). Moreover, it could effectively inhibit the biofilm formation of *S. mutans* and significantly reduce the amount of EPS produced by the biofilm (*p* < 0.01)	[74]
*Salvia sclarea*	Greece	Methanol	The extract at MIC: 0.08–2.50 mg/mL showed high antibacterial activity	[61]
*Schinus terebinthifolius*	Brazil	Water–alcohol fraction, DMSO	The adhesion of *S. mutans* biofilms was best inhibited by the extract and its methanol fraction (*p* < 0.05)	[62]
*Symplocarpus renifolius*	Korea	Ethanol	*S. renifolius* extract significantly repressed the gtf and spaP genes for glucan synthesis and adhesive proteins in *S. mutans*	[75]
*Thuja orientalis*	Korea	Methanol	The extract (1.0 mg/mL) significantly inhibited bacterial growth by more than 83%	[57]
*Thymus longicaulis*	Greece	Methanol	Biofilm formation inhibited by more than 98.0% using the same concentration	[61]
*Vitis coignetiae*	Japan	Juice	The extract reduced *S. mutans* adhesion to hydroxyapatite granules and biofilm formation in a dose-dependent manner. The MIC was 7.50 mg/mL. The extract inhibited gtfB activity associated with the synthesis of water-soluble glucans. It also inhibited gtfD activity associated with the synthesis of water-soluble glucans at a concentration that was lower than that used to inhibit gtfB	[76]
*Uvaria chamae*	Nigeria	Chloroform	The chloroform extract of *U. chamae* from Nigeria exhibited an MIC of 0.02 mg/mL. After 6 h of exposure, the extract at concentrations of 0.005, 0.01 and 0.02 mg/mL significantly reduced the *S. mutans* adhesion by 39%, 59% and 77%, respectively (*p* < 0.05). Furthermore, *U. chamae* notably inhibited acid production in *S. mutans* at 10, 12, 14 and 16 h (*p* < 0.05). At half the MIC, the extract significantly downregulated virulence genes involved in adhesion, biofilm formation and extracellular matrix production	[77]
*Withania somnifera*	Nepal	Methanol	The extract demonstrated a broad antibacterial spectrum against oral bacteria (MIC: 0.125–2 mg/mL). At levels below MIC, dose-dependent methanolic extract increased the doubling time of *S. mutans* by up to 258%; the extract inhibited acid generation, acid resistance and biofilm formation in *S. mutans* at levels below MIC	[78]
*Zīngiber officināle*	India	Methanol	Biofilm formation in *S. mutans* was reduced during critical growth phases; glucan synthesis and adhesion were suppressed. The qRT-PCR data showed downregulation of virulence genes, which is believed to be one of the main reasons responsible for the observed decrease in virulence properties. The treated rats showed an incredible reduction in caries development compared to the untreated group	[79]

## Data Availability

No new data were created or analyzed in this study.

## Abbreviations

ATCC	
atpD	beta ATP synthase
BHI	brain heart infusion
CFU	colony-forming unit
CLSM	confocal laser scanning microscopy
CHX	chlorhexidine
DIZ	diameter of the inhibition zone
DMSO	dimethyl sulfoxide
DNA	deoxyribonucleic acid
EO	essential oil
EPS	extracellular polysaccharide
gtf	glucosyltransferase
ldh	lactate dehydrogenase
MBC	minimal bactericidal concentration
MBEC	minimum biofilm eradication concentration
MIC	minimal inhibitory concentration
OD	optical density
PCR	polymerase chain reaction
QS	quorum sensing
TBH	todd hewitt broth
TSB	tryptic soy broth
SFE	exopolysaccharides;
XTT	2,3-bis(2-methoxy-4-nitro-5-sulphophenyl)-5-[(phenyl amino) carbonyl]-2H-tetrazolium hydroxide assay
